# Supplementary figures and images for: The SpxA1-TenA toxin-antitoxin system regulates epigenetic variations of Streptococcus pneumoniae by targeting protein synthesis
Source: PLoS Pathog. 2024 Dec 26;20(12):e1012801. doi: 10.1371/journal.ppat.1012801 (PMC11709252; doi:10.1371/journal.ppat.1012801)

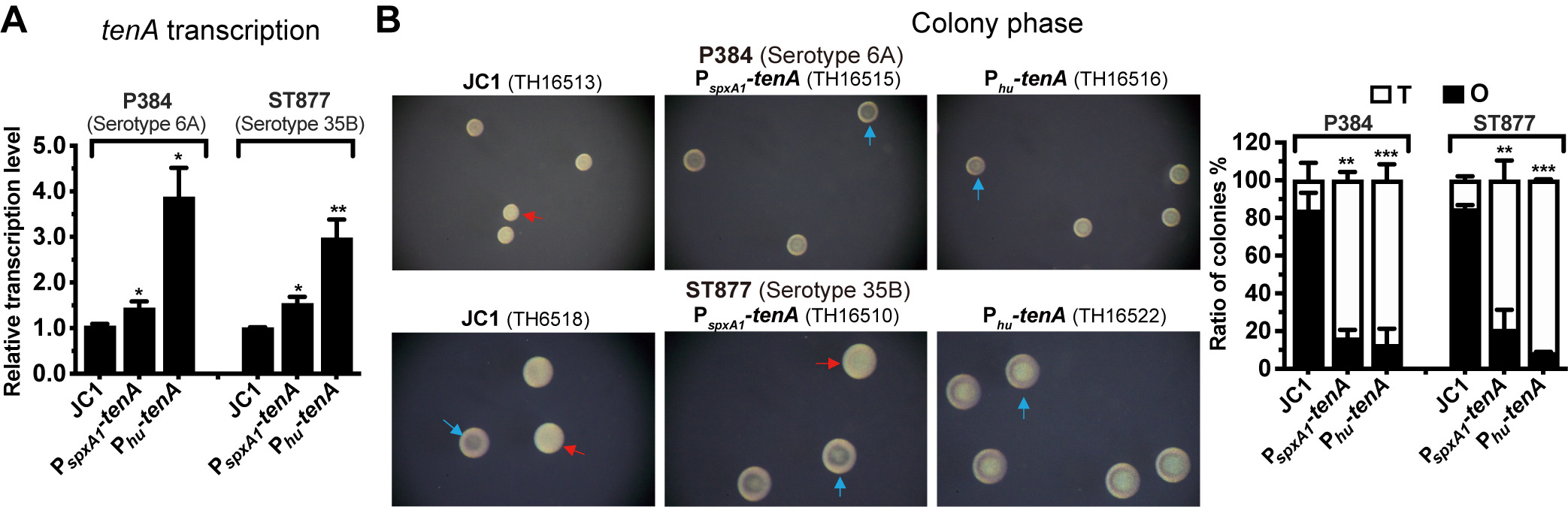

Supplement: S1 Fig — (A) Relative transcriptional expression of tenA in tenA-overexpressed derivatives of strains P384 (serotype 6A) and ST877 (serotype 35B) were detected and presented as in Fig 1D. (B) Colony phenotypes and ratio between O and T colonies in the tenA-overexpressed derivatives of strains P384 and ST877. (TIF) [file ppat.1012801.s001.tif]

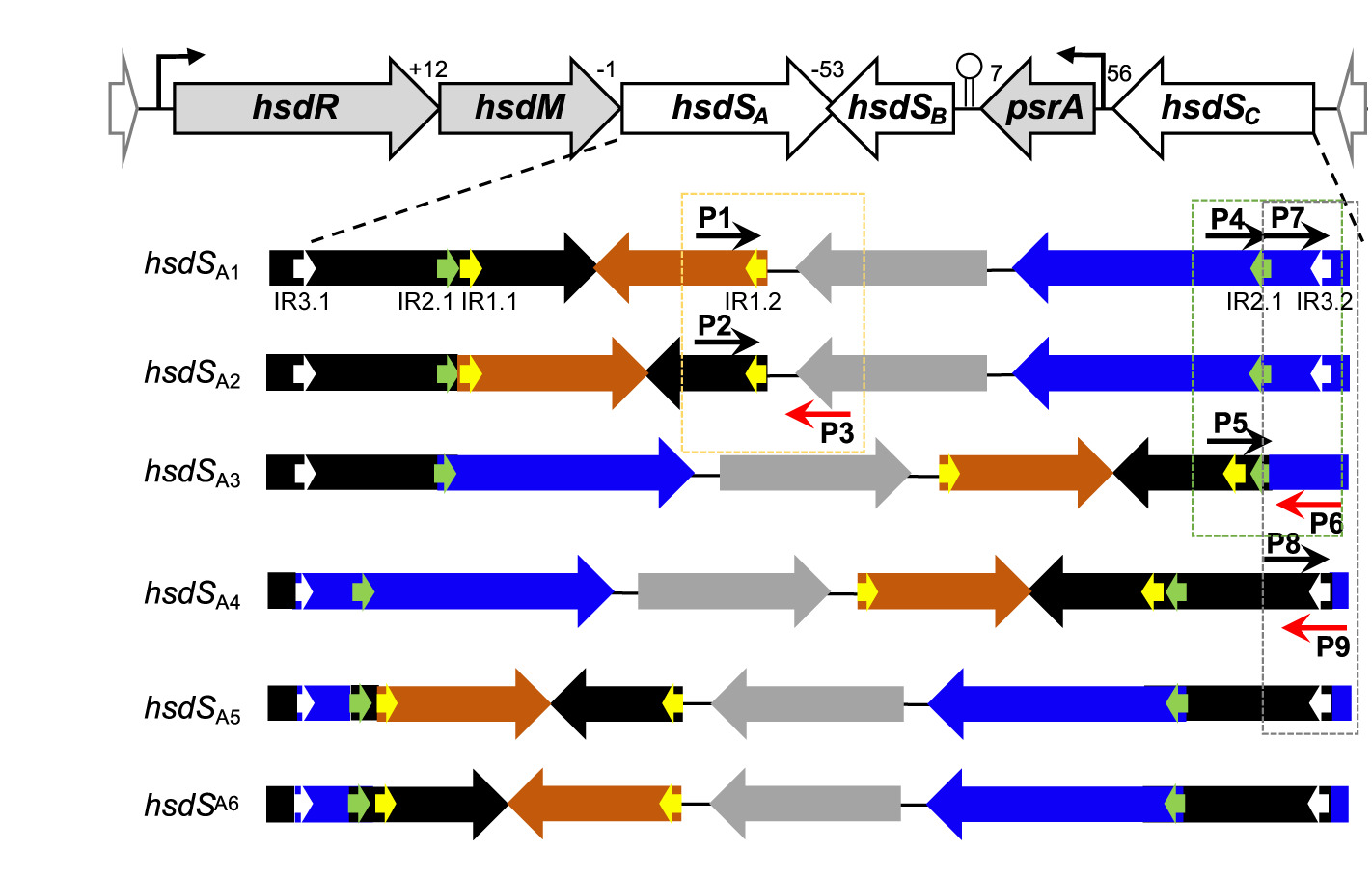

Supplement: S2 Fig — The genes encoding the restriction enzyme (hsdR), DNA methyltransferase (hsdM), sequence recognition proteins (hsdSA, hsdSB and hsdSC) and invertase (psrA) are depicted at the top. The promoter and rho-independent transcription terminator are indicated by an arrow and a hairpin. The allelic variants of the hsdSA gene are depicted below. Three pairs of inverted repeats are indicated by colored arrows (IR1: yellow, IR2: green and IR3: white). PCR primers for detecting total mRNA of hsdS (P1, P2) and hsdSA1 (P3, P4) are indicated. (TIF) [file ppat.1012801.s002.tif]

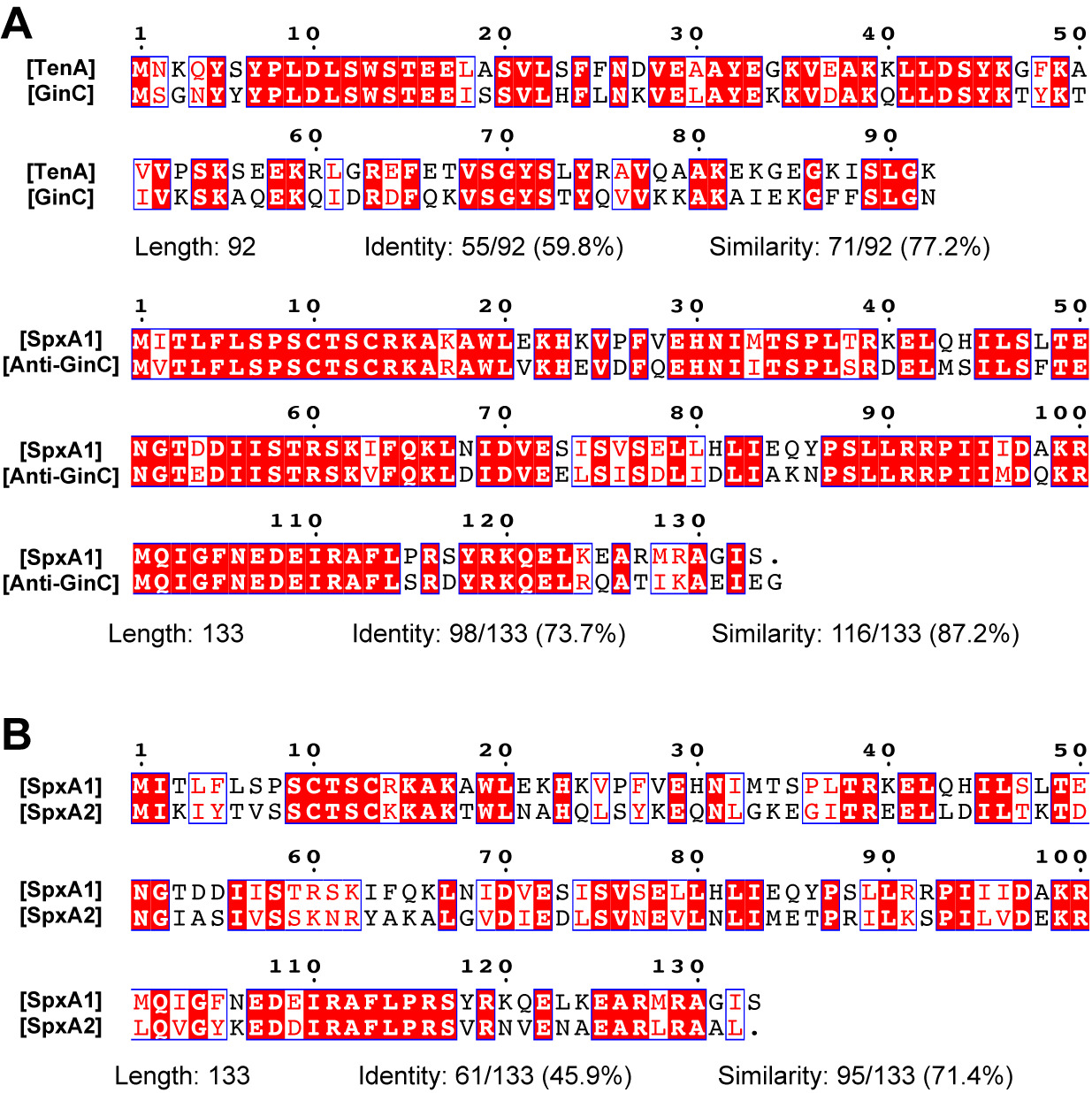

Supplement: S3 Fig — (A) Alignment between SpxA1-TenA system in S. pneumoniae and SPY_RS05235-RS05235 system in S. pyogenes. TenA in S. pneumoniae, SpxA1 in S. pneumoniae, GinC (SPY_RS05230) and Anti-GinC (SPY_RS05235) in S. pyogenes M1 (NC_002737.2) were aligned by Smith-Waterman method. Identical or similar amino acids are indicated by blue box. The black color indicates the amino acids that are not similar or the gaps. (B) Alignment between SpxA1 and SpxA2 in S. pneumoniae. SpxA1 and SpxA2 were aligned and presented as in (A). (TIF) [file ppat.1012801.s003.tif]

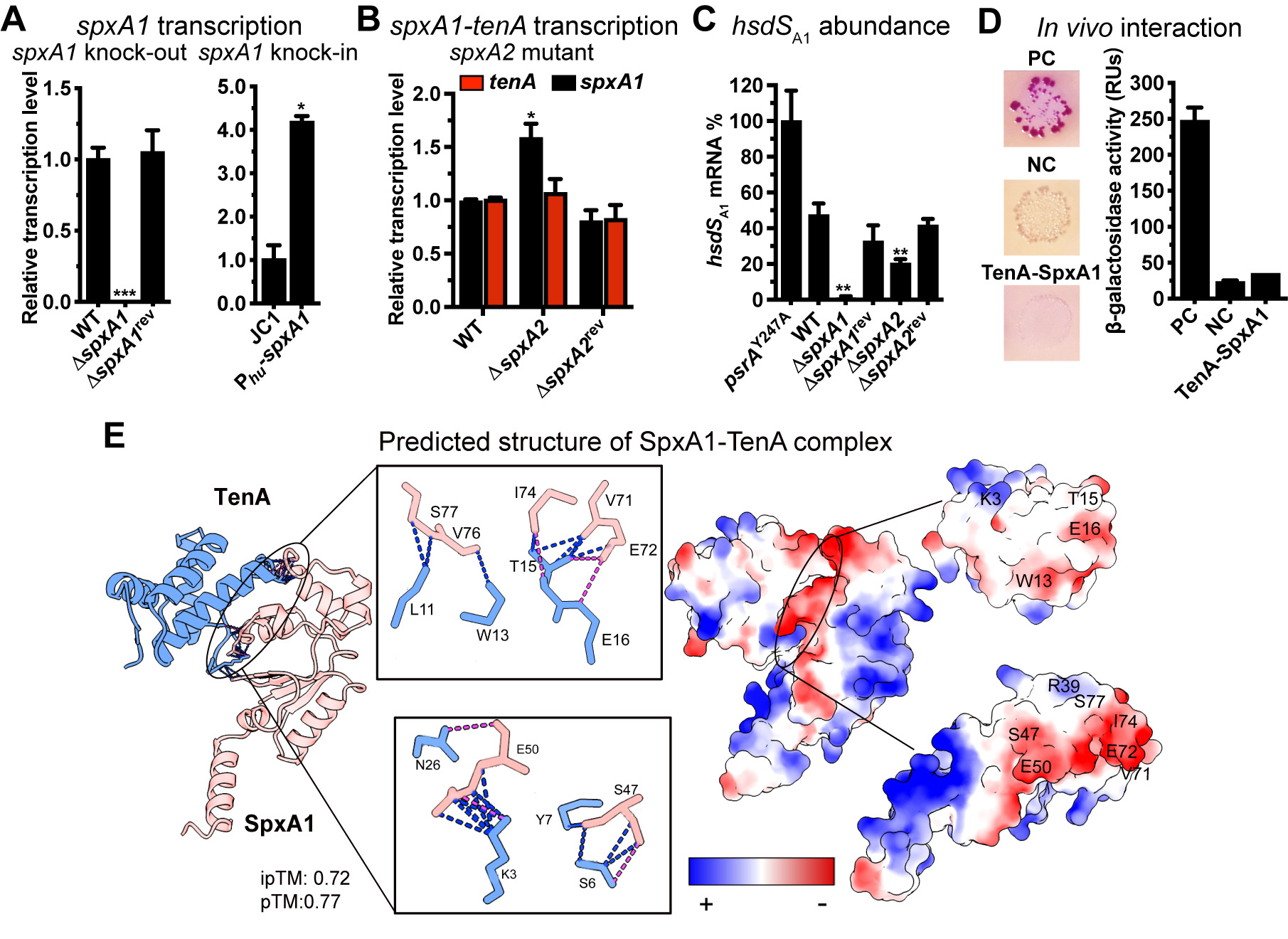

Supplement: S4 Fig — (A) The relative mRNA levels of spxA1 in the spxA1 deletion and overexpression variants were detected by RT-qPCR as in Fig 1C. The mRNA levels were presented as relative values to that of WT (left panel) and JC1 control strain (right panel), respectively. (B) The relative mRNA levels of spxA1-tenA in spxA2 mutant and revertant were detected by RT-qPCR as in Fig 1C. (C) Relative abundance of the hsdSA1 mRNA in spxA1 and spxA2 mutants. (D) Detection of TenA interactions with SpxA1 by bacterial two-hybrid (BACTH) assay. Colonies on the MacConkey/maltose plates (left panel) and β-galactosidase activity (right panel) are shown for each reporter strain. PC, positive control (pKT25-zip and pUT18C-zip). NC, negative control (empty vectors pKT25 and pUT18C). The mean ± s.d. of three values (from three individual experiments) of each strain is presented in a single bar. (E) Predicted interaction of the SpxA1-TenA complex. The structural models and surface electro statics were Predicted, presented and labelled as in Fig 6C. TenA and SpxA1 are Indicated as blue and pink. (TIF) [file ppat.1012801.s004.tif]

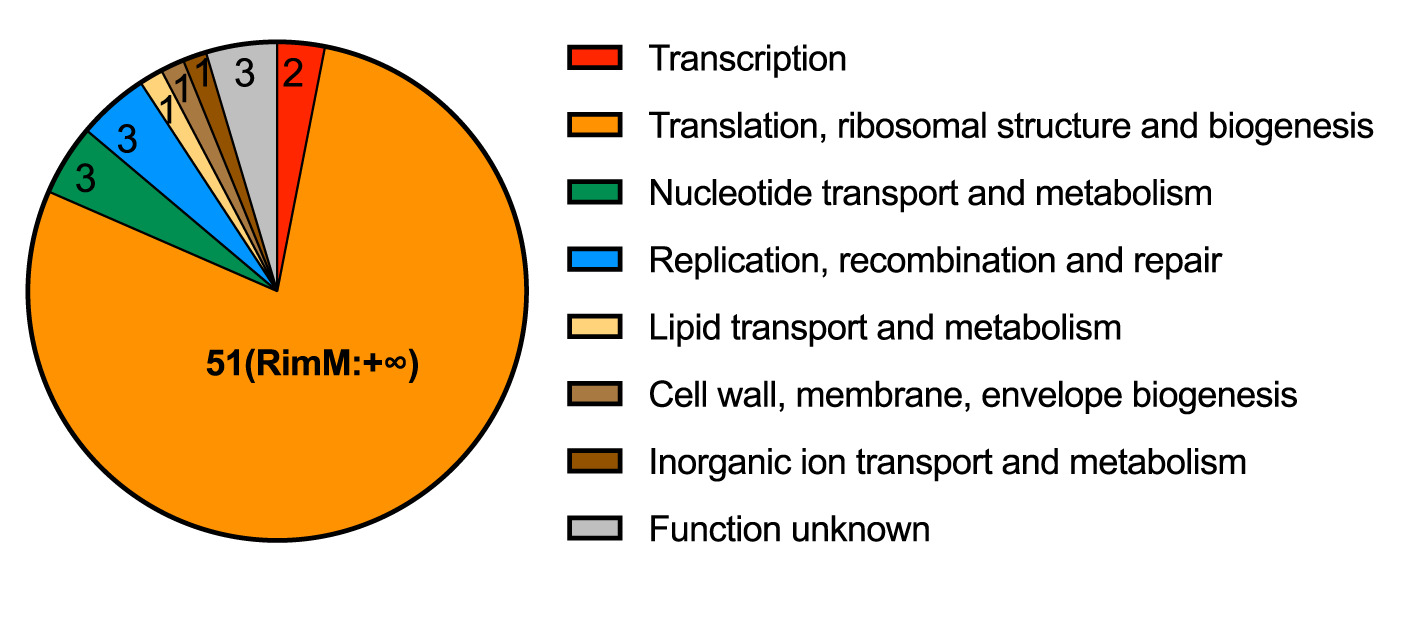

Supplement: S5 Fig — Each slice lists the numbers of proteins functions based on the assignment to molecular function categories in the Gene Ontology (GO, available at www.geneontology.org/). (TIF) [file ppat.1012801.s005.tif]

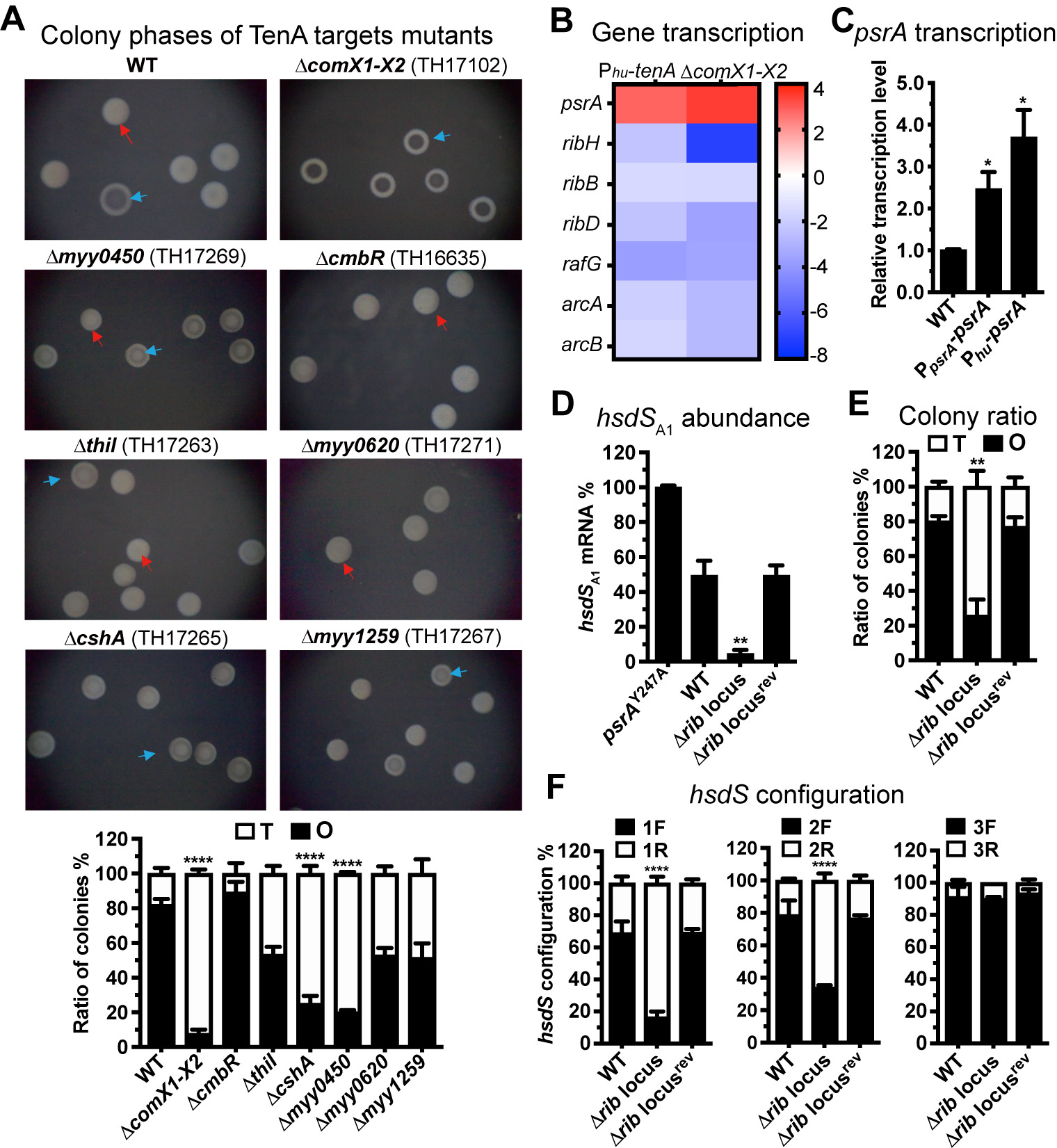

Supplement: S6 Fig — (A) Colony phenotype and ratio between O and T colonies of the TenA-targeted protein mutants. (B) Verification of the expression of the psrA, rib, rafG, and arcA locus in the tenA-overexpressed variant and comX deletion mutants. (C) Relative expression of psrA in the psrA-overexpressed variants. (D to F) Relative abundance of the hsdSA1 mRNAs (D), ratio between O and T colonies (E) and hsdSA allelic configurations (F) of the rib locus mutants. (TIF) [file ppat.1012801.s006.tif]

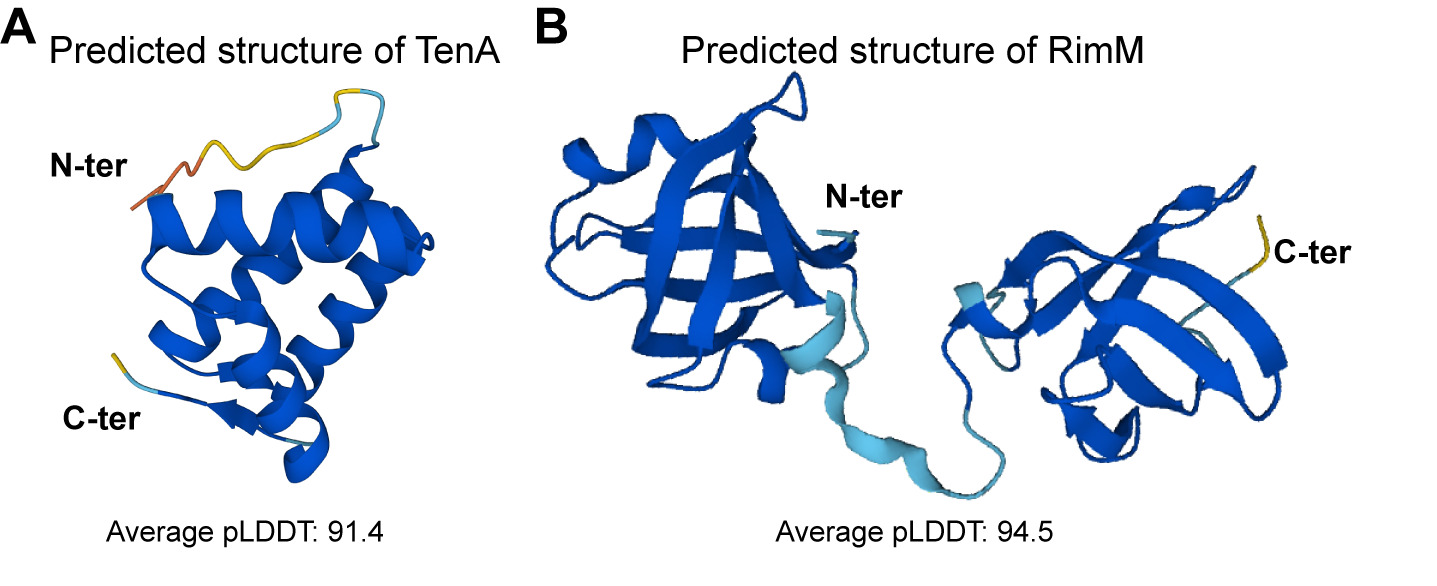

Supplement: S7 Fig — (A and B) Cartoon model of the structure of TenA (A) and RimM (B) predicted by AlphaFold3. Red (low confidence) and blue (high confidence) colors show the pLDDT values per position. The N-terminal domain (N-ter) and C-terminal domain (C-ter) of TenA and RimM are indicated. The pLDDT values are marked. (TIF) [file ppat.1012801.s007.tif]
