# Supplementary material for: The SpxA1-TenA toxin-antitoxin system regulates epigenetic variations of Streptococcus pneumoniae by targeting protein synthesis
Source: PLoS Pathog. 2024 Dec 26;20(12):e1012801. doi: 10.1371/journal.ppat.1012801 (PMC11709252; doi:10.1371/journal.ppat.1012801)
Supplement: S1 Table — (DOCX) [file ppat.1012801.s008.docx]

S1 Table. The opacity ratio of ST556 derivatives^*^

| **Strain ID (genotype)** | **Number** | | **%^†^**  **O** | **%^‡^**  **T** | **SEM (±)^§^** | **Chi-square, df** | ***P* value** | **Colony phase** |
| --- | --- | --- | --- | --- | --- | --- | --- | --- |
|  | **O** | **T** |  |  |  |  |  |  |
| ST606 ST556 *rpsL1* | 178 | 14 | 88.2 | 11.8 | 2.6 | **_** | **_** | O |
|  | 125 | 17 |  |  |  |  |  |  |
|  | 109 | 21 |  |  |  |  |  |  |
| TH9164  ∆*rr06* | 51 | 120 | 29.7 | 70.3 | 2.0 | 1.0, 1 | 5.52E-05 | T |
|  | 30 | 84 |  |  |  |  |  |  |
|  | 45 | 91 |  |  |  |  |  |  |
| TH9551  *∆rr06*^rev-N^ | 9 | 73 | 11.0 | 89.0 | 1.4 | 1.0, 1 | 1.24E-05 | T |
|  | 11 | 71 |  |  |  |  |  |  |
|  | 5 | 54 |  |  |  |  |  |  |
| TH14037  ∆ATAT | 171 | 29 | 83.3 | 16.7 | 3.5 | 0.8, 1 | 3.23E-01 | O |
|  | 124 | 17 |  |  |  |  |  |  |
|  | 65 | 20 |  |  |  |  |  |  |
| TH8198 JC1 | 49 | 7 | 86.1 | 13.9 | 0.7 | **_** | **_** | O |
|  | 52 | 9 |  |  |  |  |  |  |
|  | 47 | 8 |  |  |  |  |  |  |
| TH14084  P*_spxA1_-*full operon | 18 | 171 | 10.9 | 89.1 | 2.2 | 1.0, 1 | 5.13E-06 | T |
|  | 16 | 89 |  |  |  |  |  |  |
|  | 10 | 114 |  |  |  |  |  |  |
| TH14130  P*_spxA1_-spxA1* | 130 | 14 | 88.5 | 11.5 | 0.9 | 0.2, 1 | 9.73E-02 | O |
|  | 86 | 12 |  |  |  |  |  |  |
|  | 105 | 15 |  |  |  |  |  |  |
| TH14122 P*_spxA1_-tenA* | 20 | 106 | 15.6 | 84.4 | 2.7 | 1.0, 1 | 1.39E-05 | T |
|  | 18 | 72 |  |  |  |  |  |  |
|  | 11 | 91 |  |  |  |  |  |  |
| TH14124  P*_spxA1_-myy887* | 87 | 10 | 92.0 | 8.0 | 1.2 | 0.1, 1 | 1.21E-02 | O |
|  | 172 | 13 |  |  |  |  |  |  |
|  | 111 | 8 |  |  |  |  |  |  |
| TH14126  P*_spxA1_-myy888* | 146 | 14 | 90.4 | 9.6 | 2.9 | 0.1, 1 | 2.22E-01 | O |
|  | 108 | 19 |  |  |  |  |  |  |
|  | 92 | 5 |  |  |  |  |  |  |

S1 Table. The opacity ratio of ST556 derivative (Continued)

| **Strain ID (genotype)** | **Number** | | **%**  **O** | **%**  **T** | **SEM (±)** | **Chi-square, df** | ***P* value** | **Colony phase** |
| --- | --- | --- | --- | --- | --- | --- | --- | --- |
|  | O | T |  |  |  |  |  |  |
| TH8198  JC1 | 49 | 7 | 86.1 | 13.9 | 5.4 | **_** | **_** | O |
|  | 52 | 9 |  |  |  |  |  |  |
|  | 47 | 8 |  |  |  |  |  |  |
| TH14122  P*_spxA1_-tenA* | 20 | 106 | 15.6 | 84.4 | 2.6 | 1.0, 1 | 1.39E-05 | T |
|  | 18 | 72 |  |  |  |  |  |  |
|  | 11 | 91 |  |  |  |  |  |  |
| TH15070  P*_hu_-tenA* | 1 | 68 | 3.7 | 96.3 | 4.9 | 1.0, 1 | 7.27E-07 | T |
|  | 4 | 62 |  |  |  |  |  |  |
|  | 3 | 77 |  |  |  |  |  |  |
| TH16513 P384 JC1 | 238 | 14 | 84.0 | 16.0 | 0.7 | **_** | **_** | O |
|  | 137 | 42 |  |  |  |  |  |  |
|  | 220 | 51 |  |  |  |  |  |  |
| TH16515  P384 P*_spxA1_-tenA* | 47 | 180 | 16.2 | 83.8 | 2.7 | 1.0, 1 | 3.37E-04 | T |
|  | 31 | 232 |  |  |  |  |  |  |
|  | 43 | 223 |  |  |  |  |  |  |
| TH16516  P384 P*_hu_-tenA* | 23 | 238 | 12.8 | 87.2 | 1.4 | 1.0, 1 | 6.08E-04 | T |
|  | 18 | 237 |  |  |  |  |  |  |
|  | 59 | 202 |  |  |  |  |  |  |
| TH16518 ST877 JC1 | 148 | 22 | 84.7 | 15.3 | 1.2 | **_** | **_** | O |
|  | 118 | 24 |  |  |  |  |  |  |
|  | 126 | 24 |  |  |  |  |  |  |
| TH16520  ST877 P*_spxA1_-tenA* | 22 | 232 | 20.8 | 79.2 | 6.1 | 1.0, 1 | 5.02E-04 | T |
|  | 56 | 165 |  |  |  |  |  |  |
|  | 56 | 142 |  |  |  |  |  |  |
| TH16522  ST877 P*_hu_-tenA* | 18 | 181 | 8.4 | 91.6 | 0.3 | 1.0, 1 | 4.35E-07 | T |
|  | 11 | 120 |  |  |  |  |  |  |
|  | 11 | 128 |  |  |  |  |  |  |
| TH6552  *psrA*^Y247A^ | 129 | 3 | 98.7 | 1.3 | 0.7 | **_** | **_** | O |
|  | 133 | 2 |  |  |  |  |  |  |
|  | 121 | 0 |  |  |  |  |  |  |

S1 Table. The opacity ratio of ST556 derivative (Continued)

| **Strain ID (genotype)** | **Number** | | **%**  **O** | **%**  **T** | **SEM (±)** | **Chi-square, df** | ***P* value** | **Colony phase** |
| --- | --- | --- | --- | --- | --- | --- | --- | --- |
|  | O | T |  |  |  |  |  |  |
| TH14558 *psrA*^Y247A^  JC1 | 197 | 2 | 98.6 | 1.4 | 0.4 | 0.3, 1 | 8.45E-01 | O |
|  | 125 | 3 |  |  |  |  |  |  |
|  | 199 | 2 |  |  |  |  |  |  |
| TH14562 *psrA*^Y247A^  P*_spxA1_-tenA* | 209 | 3 | 98.8 | 1.2 | 0.4 | 0.3, 1 | 9.04E-01 | O |
|  | 228 | 1 |  |  |  |  |  |  |
|  | 175 | 3 |  |  |  |  |  |  |
| TH15321 *psrA*^Y247A^  P*_hu_-tenA* | 197 | 3 | 98.6 | 1.4 | 0.2 | 0.3, 1 | 8.27E-01 | O |
|  | 197 | 2 |  |  |  |  |  |  |
|  | 166 | 3 |  |  |  |  |  |  |
| TH15070  P*_hu_-tenA* | 15 | 186 | 10.2 | 89.8 | 1.8 | **_** | **_** | T |
|  | 21 | 200 |  |  |  |  |  |  |
|  | 24 | 154 |  |  |  |  |  |  |
| ST606 ST556 *rpsL1* | 189 | 50 | 82.0 | 18.0 | 1.9 | **_** | **_** | O |
|  | 172 | 29 |  |  |  |  |  |  |
|  | 209 | 48 |  |  |  |  |  |  |
| TH17102  ∆*comX1-X2* | 9 | 101 | 7.7 | 92.3 | 1.3 | 1.0, 1 | 5.84E-06 | T |
|  | 11 | 201 |  |  |  |  |  |  |
|  | 13 | 119 |  |  |  |  |  |  |
| TH16635  ∆*cmbR* | 99 | 21 | 89.2 | 10.8 | 3.5 | 0.1, 1 | 1.46E-01 | O |
|  | 231 | 14 |  |  |  |  |  |  |
|  | 107 | 11 |  |  |  |  |  |  |
| TH17263  ∆*thiI* | 121 | 91 | 53.3 | 46.7 | 2.6 | 1.0, 1 | 8.57E-04 | O |
|  | 212 | 178 |  |  |  |  |  |  |
|  | 179 | 191 |  |  |  |  |  |  |
| TH17265  ∆*cshA* | 30 | 91 | 25.1 | 74.9 | 2.5 | 1.0, 1 | 5.76E-05 | T |
|  | 51 | 121 |  |  |  |  |  |  |
|  | 31 | 117 |  |  |  |  |  |  |
| TH17269  ∆*myy450* | 60 | 221 | 20.2 | 79.8 | 0.6 | 1.0, 1 | 6.59E-06 | T |
|  | 47 | 190 |  |  |  |  |  |  |
|  | 47 | 195 |  |  |  |  |  |  |

S1 Table. The opacity ratio of ST556 derivative (Continued)

| **Strain ID (genotype)** | **Number** | | **%**  **O** | **%**  **T** | **SEM (±)** | **Chi-square, df** | ***P* value** | **Colony phase** |
| --- | --- | --- | --- | --- | --- | --- | --- | --- |
|  | O | T |  |  |  |  |  |  |
| TH17271  ∆*myy620* | 98 | 79 | 53.0 | 47.0 | 2.4 | 1.0, 1 | 6.83E-04 | O |
|  | 94 | 101 |  |  |  |  |  |  |
|  | 88 | 71 |  |  |  |  |  |  |
| TH17267  ∆*myy1259* | 132 | 99 | 51.5 | 48.5 | 4.8 | 1.0, 1 | 4.10E-03 | O |
|  | 79 | 109 |  |  |  |  |  |  |
|  | 111 | 89 |  |  |  |  |  |  |
| ST606 ST556 *rpsL1* | 78 | 26 | 77.0 | 23.0 | 1.2 | **_** | **_** | O |
|  | 90 | 27 |  |  |  |  |  |  |
|  | 94 | 25 |  |  |  |  |  |  |
| TH17096  ∆*comX1* | 21 | 98 | 23.1 | 76.9 | 3.2 | 1.0, 1 | 9.37E-05 | T |
|  | 27 | 90 |  |  |  |  |  |  |
|  | 37 | 92 |  |  |  |  |  |  |
| TH17099  ∆*comX2* | 11 | 91 | 8.5 | 91.5 | 2.9 | 1.0, 1 | 2.46E-05 | T |
|  | 4 | 137 |  |  |  |  |  |  |
|  | 13 | 96 |  |  |  |  |  |  |
| TH17102  ∆*comX1-X2* | 13 | 90 | 16.0 | 84.0 | 1.7 | 1.0, 1 | 7.84E-06 | T |
|  | 20 | 97 |  |  |  |  |  |  |
|  | 22 | 99 |  |  |  |  |  |  |
| TH8198  JC1 | 176 | 24 | 88.1 | 11.9 | 0.6 | **_** | **_** | O |
|  | 143 | 21 |  |  |  |  |  |  |
|  | 147 | 18 |  |  |  |  |  |  |
| TH17272  P*_psrA_-psrA* | 7 | 110 | 6.5 | 93.5 | 1.2 | 1.0, 1 | 4.22E-07 | T |
|  | 6 | 122 |  |  |  |  |  |  |
|  | 11 | 114 |  |  |  |  |  |  |
| TH17273  P*_hu_-psrA* | 5 | 96 | 4.5 | 95.5 | 1.2 | 1.0, 1 | 8.37E-07 | T |
|  | 2 | 117 |  |  |  |  |  |  |
|  | 8 | 108 |  |  |  |  |  |  |
| TH17260  P*_psrA_-psrA*^Y247A^ | 130 | 31 | 82.0 | 18.0 | 1.2 | 0.9, 1 | 6.38E-02 | O |
|  | 125 | 34 |  |  |  |  |  |  |
|  | 122 | 19 |  |  |  |  |  |  |

S1 Table. The opacity ratio of ST556 derivative (Continued)

| **Strain ID (genotype)** | **Number** | | **%**  **O** | **%**  **T** | **SEM (±)** | **Chi-square, df** | ***P* value** | **Colony phase** |
| --- | --- | --- | --- | --- | --- | --- | --- | --- |
|  | O | T |  |  |  |  |  |  |
| ST606 ST556 *rpsL1* | 70 | 15 | 80.2 | 19.8 | 1.7 | **_** | **_** | O |
|  | 60 | 18 |  |  |  |  |  |  |
|  | 65 | 15 |  |  |  |  |  |  |
| TH15908  Δ*rib* | 17 | 94 | 25.8 | 74.2 | 5.3 | 1.0, 1 | 6.08E-04 | T |
|  | 30 | 69 |  |  |  |  |  |  |
|  | 46 | 98 |  |  |  |  |  |  |
| TH16341  Δ*rib*^rev^ | 98 | 21 | 77.0 | 28.2 | 3.1 | 0.6, 1 | 4.20E-01 | O |
|  | 93 | 28 |  |  |  |  |  |  |
|  | 74 | 29 |  |  |  |  |  |  |
| TH17275  (AT-10) | 152 | 42 | 83.0 | 17.0 | 2.3 | 0.1,1 | 1.08E-01 | O |
|  | 176 | 27 |  |  |  |  |  |  |
|  | 158 | 31 |  |  |  |  |  |  |
| TH17276  (AT-18) | 125 | 32 | 78.0 | 22.0 | 0.9 | **_** | **_** | O |
|  | 105 | 32 |  |  |  |  |  |  |
|  | 101 | 29 |  |  |  |  |  |  |
| TH9551  *∆rr06*^rev-N^ (AT-22) | 29 | 149 | 15.2 | 87.8 | 4.2 | 1.0, 1 | 1.26E-04 | T |
|  | 13 | 160 |  |  |  |  |  |  |
|  | 33 | 118 |  |  |  |  |  |  |
| TH17277  (AT-38) | 18 | 138 | 14.9 | 85.1 | 2.0 | 1.0, 1 | 8.89E-06 | T |
|  | 20 | 116 |  |  |  |  |  |  |
|  | 27 | 119 |  |  |  |  |  |  |

^*^the opaque and transparent colonies on each plate in a representative experiment were enumerated as described in Fig 1.

^†, ‡^the average opaque and transparent colony ratio were calculated by the average of followed values from three plates: number of each form of colony divided by the total colony number *100%.

^§^The standard error of means was calculated on the basis of the three duplicate plates.
