# Supplementary material for: The SpxA1-TenA toxin-antitoxin system regulates epigenetic variations of Streptococcus pneumoniae by targeting protein synthesis
Source: PLoS Pathog. 2024 Dec 26;20(12):e1012801. doi: 10.1371/journal.ppat.1012801 (PMC11709252; doi:10.1371/journal.ppat.1012801)
Supplement: S3 Table — (DOCX) [file ppat.1012801.s010.docx]

S3 Table. Methylation sequences specified by the Spn556III MTase^*^

| **Genotype** | **HsdS_1_** | | | **HsdS_2_** | | | **HsdS_3_** | | |
| --- | --- | --- | --- | --- | --- | --- | --- | --- | --- |
|  | **5’-GAT^m6^AN_7_TCA-3’**  **3’-CTATN_7_^m6^AGT-5’** | | | **5’-GG^m6^AN_7_TGA-3’**  **3’-CCTN_7_^m6^ACT-5’** | | | **5’-GG^m6^AN_7_TCA-3’**  **3’-CCTN_7_^m6^AGT-5’** | | |
|  | # in  genome^†^ | #  detected^‡^ | %  detected^§^ | # in genome | # detected | % detected | # in genome | # detected | % detected |
| Wild type | 1246 | 1246 | 100 | 2244 | 0 | 0 | 2080 | 0 | 0 |
| P*_hu_-tenA* | 1246 | 1246 | 100 | 2244 | 0 | 0 | 2080 | 0 | 0 |
| ∆*comX1-X2* | 1246 | 1246 | 100 | 2244 | 0 | 0 | 2080 | 0 | 0 |

^*^The accumulative number of all methylated loci in each strain exceeded 100% because a base was considered as being methylated once more than 30% of all the reads at the position passed the cutoff value in the PacBio platform.

^†^Total number of loci in both DNA strands in the genome of ST556 (accession CP003357.2).

^‡^Total loci detected by the SMRT sequencing.

^§^Percentage of the detected motifs was calculated as follows: total loci detected/total loci in the genome.

N, any nucleotide.
