# Supplementary material for: The SpxA1-TenA toxin-antitoxin system regulates epigenetic variations of Streptococcus pneumoniae by targeting protein synthesis
Source: PLoS Pathog. 2024 Dec 26;20(12):e1012801. doi: 10.1371/journal.ppat.1012801 (PMC11709252; doi:10.1371/journal.ppat.1012801)
Supplement: S7 Table — (DOCX) [file ppat.1012801.s014.docx]

**S7** **Table. Bacterial strains or plasmids used in this study**

| **Strain ID** | **Description** | **Source** |
| --- | --- | --- |
| Strains | | |
| ST556 | *Streptococcus pneumoniae* serotype 19F | (*1*) |
| ST606 | ST556 derivative; *rpsL1* | (*2*) |
| TH9164 | TH9114 derivative; ∆*rr06* | (*3*) |
| TH9551 | TH9115 derivative; ∆*rr06*^rev-N^ | (*3*) |
| TH6552 | TH5993 derivative; *psrA*^Y247A^ | (*2*) |
| TH6671 | P384 derivative; *rpsL1* | (*2*) |
| TH6675 | ST877 derivative; *rpsL1* | (*2*) |
| TH8198 | ST606 derivative; JC1 | (*4*) |
| TH14035 | TH9551 derivative; ∆ATAT*-*JC1 | This study |
| TH14037 | TH14035 derivative; ∆ATAT | This study |
| TH14084 | TH8198 derivative; P*_spxA1_-*full operon | This study |
| TH14130 | TH8198 derivative; P*_spxA1_-spxA1* | This study |
| TH14122 | TH8198 derivative; P*_spxA1_-tenA* | This study |
| TH14124 | TH8198 derivative; P*_spxA1_-myy887* | This study |
| TH14126 | TH8198 derivative; P*_spxA1_-myy888* | This study |
| TH14558 | TH6552 derivative; JC1 | This study |
| TH14562 | TH14558 derivative; P*_spxA1_-tenA* | This study |
| TH14605 | *E. coli* BL21-DE3 carrying pTH9726; Cm^R^ | This study |
| TH14607 | *E. coli* BL21-DE3 carrying pTH14588; Cm^R^ | This study |
| TH14609 | *E. coli* BL21-DE3 carrying pTH14590; Cm^R^ | This study |
| TH14611 | *E. coli* BL21-DE3 carrying pTH14592; Cm^R^ | This study |
| TH15070 | TH8198 derivative; P*_hu_-tenA* | This study |
| TH15321 | TH14558 derivative; P*_hu_-tenA* | This study |
| TH15675 | ST606 derivative; ∆*spxA1::*JC1 | This study |
| TH15679 | ST606 derivative; *spxA2::*JC1 | This study |
| TH15683 | TH15675 derivative; ∆*spxA1* | This study |
| TH15691 | TH15675 derivative; ∆*spxA1*∆*tenA* | This study |
| TH15894 | ST606 derivative; *rib::*JC1 (*myy248-251*) | This study |
| TH15908 | TH15894 derivative; ∆*rib* (*myy248-251*) | This study |
| TH16341 | TH15894 derivative; ∆*rib*^rev^ (*myy248-251*) | This study |
| TH16513 | TH6671 derivative; JC1 | This study |

S7 Table: Bacterial strains or plasmids used in this study (Continued)

| **Strain ID** | **Description** | **Source** |
| --- | --- | --- |
| TH16515 | TH16513 derivative; P*_spxA1_-tenA* | This study |
| TH16516 | TH16513 derivative; P*_hu_-tenA* | This study |
| TH16518 | TH6675 derivative; JC1 | This study |
| TH16520 | TH16518 derivative; P*_spxA1_-tenA* | This study |
| TH16522 | TH16518 derivative; P*_hu_-tenA* | This study |
| TH16571 | ST606 derivative; ∆*spxA2* | This study |
| TH16586 | *E. coli* BL21 carrying pET28a-*tenA*; Kan^R^ | This study |
| TH16635 | TH16637 derivative; ∆*cmbR* | This study |
| TH16637 | ST606 derivative; *cmbR::*JC1 | This study |
| TH17095 | ST606 derivative; ∆*comX1::*JC1 | This study |
| TH17096 | ST606 derivative; *∆comX1* | This study |
| TH17098 | ST606 derivative; ∆*comX2::*JC1 | This study |
| TH17099 | ST606 derivative; *∆comX2* | This study |
| TH17101 | ST606 derivative; ∆*comX1-X2::*JC1 | This study |
| TH17102 | ST606 derivative; ∆*comX1-X2* | This study |
| TH17255 | ST606 derivative; *∆clpP::*JC1 | This study |
| TH17256 | ST606 derivative; *∆clpP* | This study |
| TH17257 | ST606 derivative; *∆clpP*∆*comX2::*JC1 | This study |
| TH17258 | ST606 derivative; *∆clpP*∆*comX2::comX^V5H6^* | This study |
| TH17259 | ST606 derivative; ∆*rimM::*JC1 | This study |
| TH17260 | ST606 derivative; P*_psrA_-psrA*^Y247A^ | This study |
| TH17261 | ST606 derivative; *∆clpP*∆*comX2::comX^V5H6^*∆*rimM::*JC1 | This study |
| TH17262 | ST606 derivative; ∆*thiI::*JC1 | This study |
| TH17263 | ST606 derivative; ∆*thiI* | This study |
| TH17264 | ST606 derivative; ∆*cshA::*JC1 | This study |
| TH17265 | ST606 derivative; ∆*cshA* | This study |
| TH17266 | ST606 derivative; ∆*myy1259::*JC1 | This study |
| TH17267 | ST606 derivative; ∆*myy1259* | This study |
| TH17268 | ST606 derivative; ∆*myy450::*JC1 | This study |
| TH17269 | ST606 derivative; ∆*myy450* | This study |
| TH17270 | ST606 derivative; ∆*myy620::*JC1 | This study |
| TH17271 | ST606 derivative; ∆*myy620* | This study |

Table S7 Table: Bacterial strains or plasmids used in this study (Continued)

| **Strain ID** | **Description** | **Source** |
| --- | --- | --- |
| TH17272 | TH8198 derivative; P*_psrA_-psrA* | This study |
| TH17273 | TH8198 derivative; P*_hu_-psrA* | This study |
| TH17405 | TH8198 derivative; P*_hu_*-*spxA1* | This study |
| TH17274 | ST606 derivative; AT*::*JC1 | This study |
| TH17275 | ST606 derivative; 10-AT | This study |
| TH17276 | ST606 derivative; 18-AT | This study |
| TH17277 | ST606 derivative; 38-AT | This study |
| Plasmids | | |
| pIB166 | *E. coli* - *S. pneumoniae* shuttle vector, Cm^R^ | (*5*) |
| pET28a | *E. coli* protein expression vector, KanR | Novagen |
| pTH9726 | pIB166-P*_lac_*, Cm^R^ | (*6*) |
| pTH14588 | pIB166-*spxA1*; Cm^R^ | This study |
| pTH14590 | pIB166-*tenA*; Cm^R^ | This study |
| pTH7522 | pUT18C | This study |
| pTH7523 | pKT25 | This study |
| pTH7482 | pUT18C-Zipper | This study |
| pTH7485 | pKT25-Zipper | This study |
| pTH17279 | pUT18C-TenA | This study |
| pTH17280 | pUT18C-ComX | This study |
| pTH17282 | pKT25-RimM | This study |
| pTH17284 | pKT25-ComX | This study |
| pTH16586 | pET28a-*tenA* (N-terminus fused 6*his); Kan^R^ | This study |

Cm^R^: chloramphenicol resistance; *kan^R^*: kanamycin resistant gene

**References**

1. G. L. Li *et al.*, Complete genome sequence of strain ST556, a multidrug-resistant isolate from an otitis media patient. *J Bacteriol* **194**, 3294-3295 (2012).

2. J. Li *et al.*, Epigenetic switch driven by DNA inversions dictates phase variation in *Streptococcus pneumoniae*. *PLoS Pathog* **12**, e1005762 (2016).

3. J. Wang *et al.*, Regulation of pneumococcal epigenetic and colony phases by multiple two-component regulatory systems. *PLoS Pathog* **16**, e1008417 (2020).

4. X. Liu *et al.*, Transcriptional pepressor PtvR regulates phenotypic tolerance to vancomycin in *Streptococcus pneumoniae*. *J Bacteriol* **199**, e00054-00017 (2017).

5. I. Biswas, J. K. Jha, N. Fromm, Shuttle expression plasmids for genetic studies in *Streptococcus mutans*. *Microbiology (Reading)* **154**, 2275-2282 (2008).

6. Y. Liu *et al.*, HtrA-mediated selective degradation of DNA uptake apparatus accelerates termination of pneumococcal transformation. *Mol Microbiol* **112**, 1308-1325 (2019).
