# Supplementary material for: The SpxA1-TenA toxin-antitoxin system regulates epigenetic variations of Streptococcus pneumoniae by targeting protein synthesis
Source: PLoS Pathog. 2024 Dec 26;20(12):e1012801. doi: 10.1371/journal.ppat.1012801 (PMC11709252; doi:10.1371/journal.ppat.1012801)
Supplement: S8 Table — (DOCX) [file ppat.1012801.s015.docx]

**S8 Table. List of primers used in this study**

| **Primer** | **Sequence (5’-3’)** |
| --- | --- |
| Pr1098 | GAGACTCGAGCCTTTCCTTATGCTTTTGGAC |
| Pr8227 | ATCGCTGGTCGTGCTGAAATCTG |
| Pr8229 | GAGACTCGAGTCTAGAGCAAGCCAAGCAGCAGTAGCAG |
| Pr8228 | GAGATCTAGAACAAGTCCCAATCATTACTGAGCAAGC |
| Pr8230 | TCAGTTCCTTCTTACCACAAGACCATCTC |
| Pr9840 | GAGATCTAGAGGATAATGCTGAAAACTCCTTGAAG |
| Pr10454 | GGAAGAGGAAATCATCGAGAAAGAACG |
| Pr10457 | TAGTCTCGCAGAGTCCAGCCAATG |
| Pr12368 | GAGAGGGCCCGCTCTAGACGGTGATCAACACG |
| Pr15751 | TCATTATGGACAAGGCGGTTGA |
| Pr15752 | GCTCTAGAAAAACATCTTTCCTTTCACTTCCTA |
| Pr15753 | GAGACTCGAGCCAATTGTGTCAATCGTTCGCAAAA |
| Pr15754 | TTACTTTCCAAGAGAAATCTTT |
| Pr15837 | GCTCTAGAAAGGAAAGATGTTTTTAATTTACAT |
| Pr15841 | GCGCTCGAGTCATTTGAATCTGAGGCCTTTTGGA |
| Pr15853 | GCGCTCGAGTTAACTAATACCAGCTCTCATTCTT |
| Pr15854 | GTATAGATAAGAAGAGAAAAGCATGAACAAACAGTATAGTTA |
| Pr15855 | TAACTATACTGTTTGTTCATGCTTTTCTCTTCTTATCTATAC |
| Pr15856 | GCGCTCGAGTTACTTTCCAAGAGAAATCTTTCC |
| Pr15857 | GTATAGATAAGAAGAGAAAAGCTTGGAAAGTAAATTTGAATTTG |
| Pr15858 | CAAATTCAAATTTACTTTCCAAGCTTTTCTCTTCTTATCTATAC |
| Pr15859 | GCGCTCGAGTTAAGTTCTTTTCCTTTCGTAAATA |
| Pr15860 | CAAATCCTTCTGGAAATTTCATGCTTTTCTCTTCTTATCTATAC |
| Pr15861 | GTATAGATAAGAAGAGAAAAGC ATGAAATTTCCAGAAGGATTTG |
| Pr16117 | TGGTCTGCACCATTTCGCAG |
| Pr16118 | GCTCTAGAGCTTTTCTCTTCTTATCTAT |
| Pr16119 | CCGCTCGAGTCTAGAATGAACAAACAGTATAGTTA |
| Pr16120 | GTTTGAGTAATGGCATCAGAAAAGG |
| Pr16196 | ACAGCTCGGTGATAGAAATAGTGTAATCATAGATCCTTTCTCCTCTTTAGATCTTTTG |
| Pr16197 | CAAAAGATCTAAAGAGGAGAAAGGATCTATGATTACACTATTTCTATCACCGAGCTGT |
| Pr16198 | GAGACTCGAGCTAGTGATGGTGATGGTGATGACTAATACCAGCTCTCATTCTTGCTTCTTT |
| Pr16199 | ATCTAGTGGGTAACTATACTGTTTGTTCATAGATCCTTTCTCCTCTTTAGATCTTTTG |

S8 Table: List of primers used in this study (Continued)

| **Primer** | **Sequence (5’-3’)** |
| --- | --- |
| Pr16200 | CAAAAGATCTAAAGAGGAGAAAGGATCTATGAACAAACAGTATAGTTACCCACTAGAT |
| Pr16201 | GAGACTCGAGCTAGTGATGGTGATGGTGATGCTTTCCAAGAGAAATCTTTCCTTCCCCTTT |
| Pr17720 | GCTCTAGATGAAAAGCCAATTTCTGTTT |
| Pr17721 | TAACTATACTGTTTGTTCATGTTAATGATTCCTCCAAATA |
| Pr17722 | TATTTGGAGGAATCATTAACATGAACAAACAGTATAGTTA |
| Pr17787 | GGCAGAGATTGCAGCGATTCG |
| Pr17788 | GCTCTAGAGTACCTACCCCTTTCGCTACATGT |
| Pr17793 | CCGCTCGAGTCTAGAACATCAAGGCTGGGAGCACTTC |
| Pr17794 | ACCCTTATCGTTCAACGAAGCATAAACA |
| Pr17996 | CCTTTAGCAAATTCAAATTTACTTTCCAAGCTTTTCTCTTCTTATCTATACTTTGCTAC |
| Pr17997 | GTAGCAAAGTATAGATAAGAAGAGAAAAGCTTGGAAAGTAAATTTGAATTTGCTAAAGG |
| Pr18286 | TACTACCTTGCCTTCCAAGT |
| Pr18287 | CCGCTCGAGTCTAGACTCACTACCTCCAAAAAAAT |
| Pr18288 | GCTCTAGAAACAGACTCTACTTTTAGAC |
| Pr18289 | TTGACCAGAGCCAGCAGCAA |
| Pr18650 | GGAATTCCATATGAACAAACAGTATAGTTACCCAC |
| Pr18651 | CCGCTCGAGTTACTTTCCAAGAGAAATCTTTCCTTCC |
| Pr19391 | AACCTGCGACCGTTCGCTAGAGTTTGTAGAAACGCAAAAAGGC |
| Pr19392 | GCCTTTTTGCGTTTCTACAAACTCTAGCGAACGGTCGCAGGTT |
| Pr19393 | GTTTTTGGCGGATCTCATAATAAAATCTCCTAAAATGTTTTTTCTTGTAAGCTAAC |
| Pr19394 | AACATTTTAGGAGATTTTATTATGAGATCCGCCAAAAACATAAAGAAAGG |
| Pr19417 | GCAGAAGCATTATTGAAATACGAAACA |
| Pr19418 | CAAACTGTACGTATTATTGATGGTGCTTTTG |
| Pr19419 | CCGCTCGAGAGCCGCTTTCGCTTGTCA |
| Pr19420 | CTCTAGACTCGAGGTGATATACTAATATAGTTGTCACTTGAGAGAAGC |
| Pr19421 | CACCTAGCACTCATCGTTTACAGC |
| Pr19422 | GGTCGTGAAGCAATCTTGAAAGTTCAC |
| Pr19423 | GGAATACACAGCTTTCTTTGTAGTAATTATTTACATTTTTGAC |
| Pr19424 | CCGCTCGAGAATTTTCCCCTTTCTAGATACAGTCTAACAGATTAGA |
| Pr19425 | GCTCTAGACTCGAGTAAGTCATGCAAAAAAAATGAAAAAAATTAGAAAAAGTAGTTG |
| Pr19426 | CGTCCATTGCCGAAGATTCCC |
| Pr19435 | CTGAGGTACTTCATCTACCTTTTACTGGG |

S8 Table: List of primers used in this study (Continued)

| **Primer** | **Sequence (5’-3’)** |
| --- | --- |
| Pr19436 | CCGCTCGAGCAGAAATAGACAGACTCGGCTGACTAAC |
| Pr19437 | GCTCTAGACTCGAGCCGCATGGTCTATGTTAAACGTGAAG |
| Pr19438 | TACTCTAATACCTCTGGTTCTGGCACA |
| Pr19648 | GGAAAAATTGAAGTTATTAATCACCCACTGATTCAAC |
| Pr19649 | CCGCTCGAGTCATTTCTCCTTTTGAGTTTTAATTTTGTTGGTCAA |
| Pr19650 | GCTCTAGACTCGAGTGATGATAGAAGGCAAACTCGACTGG |
| Pr19651 | CGCAGCTGCAGTCGCACCA |
| Pr19652 | GCTCTAGATACAGTCTAACAGATTAGAAAACACA |
| Pr19653 | GCTCTAGAAAGGGGAAAATTATGATTAAAGAATTGTATGAAGAAGTCCAAG |
| Pr19654 | CCGCTCGAGTGCGGCCGCCTA |
| Pr19655 | AAGGGCAATTCGAAGCTTGAAGGTAAGCCTATCCCTA |
| Pr19656 | CATTAATGTATATCTCCTTCTTAAAGTTAAACAAAATTATTTC |
| Pr19657 | AGAAGGAGATATACATTAATGATTAAAGAATTGTATGAAGAAGTCCA |
| Pr19658 | TTCAAGCTTCGAATTGCCCTTGTGGGTACGGATAGTAAACTCCTTAAACAC |
| Pr19659 | GTTGCTTATCAAAAGCAAAAGAAAGTGCT |
| Pr19660 | CAAGGAGTTTTCAGCATTATCCTCTAGATTCCCAACATTAAAGTAGTTCATCTTGTCTC |
| Pr19661 | GAGACAAGATGAACTACTTTAATGTTGGGAATCTAGAGGATAATGCTGAAAACTCCTTG |
| Pr19662 | TGGAATATCAACATTGAGAACCACTGGTGAGACTCGAGCACAGAGTTTGTAGA |
| Pr19663 | TCTACAAACTCTGTGCTCGAGTCTCACCAGTGGTTCTCAATGTTGATATTCCA |
| Pr19664 | AGGTTTCTCCTTTATTCTTTGTTTTCTTTGATTTCTG |
| Pr19665 | TTGTTTGATATTGCCATCCATCAAGGT |
| Pr19666 | TTCAGCATTATCCTCTAGATTCAGAAAATCCAAAAGAATAGCGAAAATCAGT |
| Pr19667 | TCTTTTGGATTTTCTGAATCTAGAGGATAATGCTGAAAACTCCTTGAA |
| Pr19668 | TCATGCAGTATTTTGAGACTCGAGCACAGAGTTTGTAGAAAC |
| Pr19669 | GTGCTCGAGTCTCAAAATACTGCATGAATGCTCCTATCTTACTTT |
| Pr19670 | ATAAGAAAGGCTTGGTCGATGTTGAG |
| Pr19671 | GAGCATTCATGCAGTATTTTCAGAAAATCCAAAAGAATAGCGAAAATCAGTAAAAA |
| Pr19672 | CTTTTGGATTTTCTGAAAATACTGCATGAATGCTCCTATCTTACTTTTCTAGTTTG |
| Pr19673 | GCCATGGTGACAGCAGTACG |
| Pr19674 | GCATTATCCTCTAGAGAAAAAGCGGTTCAAAATGAATCGCT |
| Pr19675 | TTGAACCGCTTTTTCTCTAGAGGATAATGCTGAAAACTCCTTGAAGG |
| Pr19676 | AGAAAGAAGAATAACGAGACTCGAGCACAGAGTTTGTAGAAAC |

S8 Table: List of primers used in this study (Continued)

| **Primer** | **Sequence (5’-3’)** |
| --- | --- |
| Pr19677 | TGTGCTCGAGTCTCGTTATTCTTCTTTCTAAAGGTGGTGCG |
| Pr19678 | GGAATGGCTGCAAATCAGGCT |
| Pr19679 | CTTTAGAAAGAAGAATAACGAAAAAGCGGTTCAAAATGAATCGCTTTTTTA |
| Pr19680 | CATTTTGAACCGCTTTTTCGTTATTCTTCTTTCTAAAGGTGGTGCGA |
| Pr19681 | CCCACGTTTAAGGGCAGTCAC |
| Pr19682 | GCATTATCCTCTAGAAGTTTGGGACAGAGTTGATTTAGGG |
| Pr19683 | ACTCTGTCCCAAACTTCTAGAGGATAATGCTGAAAACTCCTTGAAG |
| Pr19684 | GCTGGCTGAGGAGACTCGAGCACAGAGTTTGTAGAAAC |
| Pr19685 | CTCGAGTCTCCTCAGCCAGCAATGCCAAC |
| Pr19686 | GTTTGAAGAATTGACCCACCGTGATG |
| Pr19687 | AGTTGGCATTGCTGGCTGAGAGTTTGGGACAGAGTTGATTTAGGG |
| Pr19688 | CCCTAAATCAACTCTGTCCCAAACTCTCAGCCAGCAATGCCAACT |
| Pr19689 | GATATTTTTGAACAAGAGTTTGGACGTGAAGT |
| Pr19690 | TCAAGGAGTTTTCAGCATTATCCTCTAGAGTTACAGTCTTGCAAGACCTTTCTATATGT |
| Pr19691 | CATATAGAAAGGTCTTGCAAGACTGTAACTCTAGAGGATAATGCTGAAAACTCCTTGA |
| Pr19692 | TCCCTTTCTATTTTACCTCTTGCCGTGAGACTCGAGCACAGAGTTTGTAGA |
| Pr19693 | TCTACAAACTCTGTGCTCGAGTCTCACGGCAAGAGGTAAAATAGAAAGGGA |
| Pr19694 | GGCCTTGATTTGCGTTGCT |
| Pr19695 | CCTTTCTATTTTACCTCTTGCCGTGTTACAGTCTTGCAAGACCTTTCTATATGTCC |
| Pr19696 | ACATATAGAAAGGTCTTGCAAGACTGTAACACGGCAAGAGGTAAAATAGAAAGG |
| Pr19697 | TTCAGATTGCGGAGGAGAGCG |
| Pr19698 | AGTTTTCAGCATTATCCTCTAGAGATAAAATCCTTTCAATAAAAAATCCCTGCTCT |
| Pr19699 | TTTTTATTGAAAGGATTTTATCTCTAGAGGATAATGCTGAAAACTCCTTGAAGG |
| Pr19700 | GGCTCAAGAAAAAGAGACTCGAGCACAGAGTTTGTAGAAAC |
| Pr19701 | TGCTCGAGTCTCTTTTTCTTGAGCCTTGCATTTCCG |
| Pr19702 | TGTCCAGATAGCGATAAACGCG |
| Pr19703 | TGCAAGGCTCAAGAAAAAGATAAAATCCTTTCAATAAAAAATCCCTGCTCTG |
| Pr19704 | CAGAGCAGGGATTTTTTATTGAAAGGATTTTATCTTTTTCTTGAGCCTTGCATTTCCGAAAA |
| Pr19705 | AGGACCAATCGAAAAACTTCTTGG |
| Pr19706 | ATTCAAAATCTTTCAAGTTAATGATTCCTCCAAATAATTTCTAATTAACAAATATAATC |
| Pr19707 | GAAATTATTTGGAGGAATCATTAACTTGAAAGATTTTGAATTACATTTGAAGAAAGCTG |
| Pr19708 | GCTTGCTCTAGACTCGAGTTACCAATTAACAATTTTATCTACAATATTTTGTTGTTCAG |

S8 Table: List of primers used in this study (Continued)

| **Primer** | **Sequence (5’-3’)** |
| --- | --- |
| Pr19709 | ACAACAAAATATTGTAGATAAAATTGTTAATTGGTAACTCGAGTCTAGAGCAAGCCAAG |
| Pr19710 | AACAGATACAAGTCTATTTTCAAGAAGAAATCAC |
| Pr19711 | GTTTTGTACTACCCGACGCTTTACAAGTCCCAATCATTACTGAGCA |
| Pr19712 | TGCTCAGTAATGATTGGGACTTGTAAAGCGTCGGGTAGTACAAAAC |
| Pr19713 | TATTCAAAAGGGTGCCCATTTTAT |
| Pr19714 | CAAGGAGTTTTCAGCATTATCCTCTAGAGTAAATTAAAAACATCTTTCCTTTCACTTCC |
| Pr19715 | GGAAGTGAAAGGAAAGATGTTTTTAATTTACTCTAGAGGATAATGCTGAAAACTCCTTG |
| Pr19716 | GTTAATTCACTCTTAACGATGGTTTTAAATGGAGACTCGAGCACAGAGTTTGTAGA |
| Pr19721 | CACTCTTAACGATGGTTTTAAATGATATATATATATATATATATATATATATATTTATATATGTAAATTAAAAACATCTTTCCTTTCAC |
| Pr19717 | TCTACAAACTCTGTGCTCGAGTCTCCATTTAAAACCATCGTTAAGAGTGAATTAAC |
| Pr19718 | AATTATTGGTACCATCAATGGGATCAATG |
| Pr19719 | GTAAATTAAAAACATCTTTCCTTTCACTTCC |
| Pr19720 | CTCTTAACGATGGTTTTAAATGATATATATATGTAAATTAAAAACATCTTTCCTTTCAC |
| Pr19722 | CATTTAAAACCATCGTTAAGAGTGAATTAAC |
| Pr19723 | GCTCTAGAGATGAACAAACAGTATAGTTACCCACT |
| Pr19724 | CGGAATTCTTACTTTCCAAGAGAAATCTTTCCTTCCC |
| Pr19725 | GCTCTAGAGATGAACTACTTTAATGTTGGGAAAATCGTTAATACG |
| Pr19726 | CGGAATTCTCAATCTTCATCGTCTAACCCTTCTAAGATTT |
| Pr19727 | GCTCTAGAGATGATTAAAGAATTGTATGAAGAAGTCCAAGG |
| Pr19728 | CGGAATTCCTAGTGGGTACGGATAGTAAACT |
| Pr19729 | GCTCTAGAGATGAAAAAAGAATTTAATTTAATTGCAACTGTGGCAG |
| Pr19730 | CGGAATTCCTATTTTACCTCTTGCCGTTTGACACG |
| Pr19731 | GCTCTAGAGATGCAGTATTCAGAAATTATGATTCGCTACG |
| Pr19732 | GGGGTACCTTAGAGTAGATTATCAATCAAGTCATCAACTTCATCTTTT |
| Pr19733 | GCTCTAGAGATGTTACAAAAAATTTATGAGCAGATGG |
| Pr19734 | CGGAATTCTCAACAAGAAATAAAACCCCGATTCA |
| Pr19957 | TAGTGTAATCATGTTAATGATTCCTCCAAATAATTTCTAATTAACAAATATAATCATAT |
| Pr19958 | TAGAAATTATTTGGAGGAATCATTAACATGATTACACTATTTCTATCACCGAGCTGTAC |

S8 Table: List of primers used in this study (Continued)

| **Primer** | **Sequence (5’-3’)** | **Application** |
| --- | --- | --- |
| Pr7932 | GATTGCCATCATGAGTGACAAGG | Detection of *era* abundance |
| Pr7933 | AGTGTCCACTTCGCGAAGGGT |  |
| P1 | TGAGTGGCAGGAATATCCAATATGGC |  |
| P2 | ATCCACCAGCCACAACACCATCATAG | Detection of IR1-mediated inversion |
| P3 | CGAATTTATCTAAGGAAAACAGCTACTGAACAAC |  |
| P4 | CTACTGGAACAAGTTATCCTGCAATCAATG | Detection of IR2-mediated inversion |
| P5 | AAAGTTGCTTCTATTCTTATCCCTCTCCCTC |  |
| P6 | CCAACTTTCTGGTATTTCACAAGGTACTTCC |  |
| P7 | CATCCATCGTGTATTGCACCATCAAC | Detection of IR3-mediated inversion |
| P8 | TCGTTAAGTAAAGTATCCAAAACAATAAATGCTG |  |
| P9 | AAAACTTATCAGTGAAGGAAAAATCAAACGAG |  |
| Pr16178 | GACACCAGAACAACTTAAAGCAAGT | Detection of *hsdS* abundance |
| Pr16179 | CACTCCCAAGTATCAGGAATATCAT |  |
| Pr16174 | GTTGCTTCTATTCTTATCCCTCTCC | Detection of *hsdS*_A1_ abundance |
| Pr16175 | TTTTTATAACAACCCAATTCATAGGT |  |
| Pr15797 | ATTCGGCTGTCAATCGTGTTTC | Detection of *myy881* abundance |
| Pr15798 | TGACCAGGCAGAGCATAAATCA |  |
| Pr15799 | TTTATAATGAAGCGGGGCAGGA | Detection of *myy882* abundance |
| Pr15800 | CCAAGGATTCATACTTCGGTCCA |  |
| Pr15801 | ACCAATCCTGACCTCAACATCC | Detection of *myy883* abundance |
| Pr15802 | ACGTTCCAAACCTAAGTGCTCA |  |
| Pr15803 | TCCTTTCCTTGACCGAAAATGG | Detection of *spxA1* abundance |
| Pr15804 | ATAATTGGACGACGCAAAAGAC |  |
| Pr15805 | TGATGTTGAAGCTGCCTATGAAG | Detection of *tenA* abundance |
| Pr15806 | TGCTCGGTAAAGTGAATAGCCA |  |
| Pr15807 | GTGTTCGTGTCTATGGAAGTGC | Detection of *myy887* abundance |
| Pr15808 | CGGCTGTCTGGTTTGAAAATCA |  |
| Pr15809 | CTGAAGGTGGTCGTCTGGTTTA | Detection of *myy888* abundance |
| Pr15810 | CAACAAACTGACCCTCTCCCTT |  |
| Pr15811 | ACCTGTGATGATTGGACTTGGG | Detection of *myy884* abundance |
| Pr15812 | AAGGCGTGAAGAAAGAAGGTCT |  |

S8 Table: List of primers used in this study (Continued)

| **Primer** | **Sequence (5’-3’)** | **Application** |
| --- | --- | --- |
| F1 | ATGATTACACTATTTCTATCACCGAGCTGTAC | Detection of co-transcription of *spxA1-tenA* |
| F2 | TTAACTAATACCAGCTCTCATTCTTGCTTCT |  |
| F3 | ATGAACAAACAGTATAGTTACCCACTAGATTTGT |  |
| F4 | TTACTTTCCAAGAGAAATCTTTCCTTCCCC |  |
| Pr17753 | CTGTCGTTAGCACACCAAACA | Detection of *rib* abundance |
| Pr17754 | TGGGAGCTGTCATTAGAGGGA |  |
| Pr17757 | TCCTGTACCATCAATGTGTCCT |  |
| Pr17758 | TGAAACATGGAGTCGAACAGC |  |
| Pr17759 | TGGTGTCGTAACTGATGCACA |  |
| Pr17760 | ACCCAGCATGTACCCTATGT |  |
| Pr17781 | TTTGATTCGTTGCGGTGGTG | Detection of *arcA* locus abundance |
| Pr17782 | ATTGGTCACGGTATTGCGGT |  |
| Pr17783 | GCGGATTCAGCCAACGTATG |  |
| Pr17784 | GCGAGCATTTGAGTTGGGTG |  |
| Pr15892 | CATCAATGCCCTTTGGTTCT | Detection of *msmG* abundance |
| Pr15893 | TGATAATGCCGACCACGTAA |  |
| Pr19735 | AAAGCAAAAACCTGGCTCAAT | Detection of *spxA2* abundance |
| Pr19736 | TGGCATAGCGATTTTTAGACG |  |
| Pr19737 | CGTGTCCCATCAAATCTGCAAG | Detection of *psrA* abundance |
| Pr19738 | ACGAACCAATTACAGCAAGAGGA |  |
