# Supplementary material for: The SpxA1-TenA toxin-antitoxin system regulates epigenetic variations of Streptococcus pneumoniae by targeting protein synthesis
Source: PLoS Pathog. 2024 Dec 26;20(12):e1012801. doi: 10.1371/journal.ppat.1012801 (PMC11709252; doi:10.1371/journal.ppat.1012801)
Supplement: S9 Table — (DOCX) [file ppat.1012801.s016.docx]

**S9 Table.** ﻿**Information for constructions of strains in this study**

| **Strain ID** | **Genotype** | **PCR amplifications** | | | **Template**  **DNA** | **Parental strains** |
| --- | --- | --- | --- | --- | --- | --- |
|  |  | **Target sequence** | **Primers** | **Digestion/Fusion** |  |  |
| TH14035 | *∆*ATAT*::*JC1 | *myy0884*-up | Pr15751/15752 | XbaI | ST606 | TH9551 |
|  |  | *myy0884*-down | Pr15753/15754 | XhoI |  |  |
| TH14037 | *∆*ATAT | *myy0884*-full length | Pr15751/15754 | - | ST606 | TH14035 |
| TH14084 | P*_spxA1_*-full operon | *bgaA*-up | Pr8227/8228 | XbaI | ST606 | TH8198 |
|  |  | *spxA1-myy0888* | Pr15837/15841 | XbaI/XhoI |  |  |
|  |  | *bgaA*-down | Pr8229/8230 | XhoI |  |  |
| TH14122 | P*_spxA1_*-*tenA* | *bgaA*-up | Pr8227/8228 | XbaI | ST606 | TH8198 |
|  |  | P*_spxA1_* | Pr15837/15855 | Pr15837/15856 |  |  |
|  |  | *tenA* | Pr15854/15856 |  |  |  |
|  |  | *bgaA*-down | Pr8229/8230 | XhoI |  |  |
| TH14558 | *psrA*^Y247A^ JC1 | *bgaA::*JC1 | Pr8229/8230 | - | TH8198 | TH6552 |
| TH14124 | P*_spxA1_*-*myy887* | *bgaA*-up | Pr8227/8228 | XbaI | ST606 | TH8198 |
|  |  | P*_spxA1_* | Pr15837/15858 | Pr15837/15859 |  |  |
|  |  | *myy0887* | Pr15857/15859 |  |  |  |
|  |  | *bgaA*-down | Pr8229/8230 | XhoI |  |  |
| TH14126 | P*_spxA1_*-*myy888* | *bgaA*-up | Pr8227/8228 | XbaI | ST606 | TH8198 |
|  |  | P*_spxA1_* | Pr15837/15861 | Pr15837/15841 |  |  |
|  |  | *myy0888* | Pr15860/15841 |  |  |  |
|  |  | *bgaA*-down | Pr8229/8230 | XhoI |  |  |

S9 Table: Information for constructions of strains in this study (Continued)

| **Strain ID** | **Genotype** | **PCR amplifications** | | | **Template**  **DNA** | **Parental strains** |
| --- | --- | --- | --- | --- | --- | --- |
|  |  | **Target sequence** | **Primers** | **Digestion/Fusion** |  |  |
| TH14130 | P*_spxA1_*-*spxA1* | *bgaA*-up | Pr8227/8228 | XbaI | ST606 | TH8198 |
|  |  | *spxA1* | Pr15837/15853 | XbaI/XhoI |  |  |
|  |  | *bgaA*-down | Pr8229/8230 | XhoI |  |  |
| TH14562 | *psrA*^Y247A^ P*_spxA1_*-*tenA* | *bgaA::tenA* | Pr8229/8230 | - | TH14122 | TH14558 |
| TH14588 | pIB166*-spxA1* | *spxA1* | Pr16197/16198 | Pr12368/16198  ApaI/XbaI | ST606 | DH5α |
|  |  | P_lac_ | Pr12368/16196 |  | TH9726 |  |
| TH14590 | pIB166*-tenA* | *tenA* | Pr16200/16201 | Pr12368/16201  ApaI/XbaI | ST606 | DH5α |
|  |  | P_lac_ | Pr12368/16199 |  |  |  |
| TH15070 | P*_hu_*-*tenA* | *bgaA*-up | Pr8227/8228 | XbaI | ST606 | TH8198 |
|  |  | P*_hu_* | Pr17720/17721 | Pr17720/15856 |  |  |
|  |  | *tenA* | Pr17722/15856 |  |  |  |
|  |  | *bgaA*-down | Pr8229/8230 | XhoI |  |  |
| TH15675 | *spxA1::*JC1 | *spxA1*-up | Pr16117/16118 | XbaI | ST606 | ST606 |
|  |  | *spxA1*-down | Pr16119/16120 | XhoI |  |  |
| TH15683 | *∆spxA1* | *spxA1-up* | Pr16117/16118 | XbaI | ST606 | TH15675 |
|  |  | *spxA1-down* | Pr16119/16120 | XbaI |  |  |
| TH15691 | *∆spxA1∆tenA* | *spxA1-up* | Pr16117/17996 | Pr16117/16120 | ST606 | TH15675 |
|  |  | *tenA-down* | Pr17997/16120 |  |  |  |
| TH15679 | *spxA2::*JC1 | *spxA2*-up | Pr17787/17788 | XbaI | ST606 | ST606 |
|  |  | *spxA2*-down | Pr17793/17794 | XhoI |  |  |

S9 Table: Information for constructions of strains in this study (Continued)

| **Strain ID** | **Genotype** | **PCR amplifications** | | | **Template**  **DNA** | **Parental strains** |
| --- | --- | --- | --- | --- | --- | --- |
|  |  | **Target sequence** | **Primers** | **Digestion/Fusion** |  |  |
| TH16571 | ∆*spxA2* | *spxA2*-up | Pr17787/17788 | XbaI | ST606 | TH15679 |
|  |  | *spxA2*-down | Pr17793/17794 | XbaI |  |  |
| TH15321 | *psrA*^Y247A^ P*_hu_*-*tenA* | P*_hu_*-*tenA* | Pr8229/8230 | - | TH15070 | TH14558 |
| TH15894 | *∆rib::*JC1 | *rib*-up | Pr18288/18289 | XbaI | ST606 | ST606 |
|  |  | *rib*-down | Pr18286/18287 | XhoI |  |  |
| TH15908 | ∆*rib* | *rib*-up | Pr18288/18289 | XbaI | ST606 | TH15894 |
|  |  | *rib*-down | Pr18286/18287 | XbaI |  |  |
| TH16341 | ∆*rib*^rev^ | *rib*-full length | Pr18288/18287 | - | ST606 | TH15894 |
| TH16513 | P384 JC1 | *bgaA::*JC1 | Pr8229/8230 | - | TH8198 | TH6671 |
| TH16515 | P384 P*_spxA1_*-*tenA* | P*_spxA1_*-*tenA* | Pr8229/8230 | - | TH14122 | TH16515 |
| TH16516 | P384 P*_hu_*-*tenA* | P*_hu_*-*tenA* | Pr8229/8230 | - | TH15070 | TH16515 |
| TH16518 | ST877 JC1 | *bgaA::*JC1 | Pr8229/8230 | - | TH8198 | TH6675 |
| TH16520 | ST877 P*_spxA1_*-*tenA* | P*_spxA1_*-*tenA* | Pr8229/8230 | - | TH14122 | TH16518 |
| TH16522 | ST877 P*_hu_*-*tenA* | P*_hu_*-*tenA* | Pr8229/8230 | - | TH15070 | TH16518 |
| TH16586 | pET28a*-tenA* | *tenA* | Pr18650/18651 | NdeI/XhoI | ST606 | BL21(DE3) |
| TH16637 | *cmbR::*JC1 | *cmbR*-up | Pr19435/19436 | XhoI | ST606 | ST606 |
|  |  | *cmbR*-down | Pr19437/19438 | XbaI |  |  |
| TH16635 | ∆*cmbR* | *cmbR*-up | Pr19435/19436 | XhoI | ST606 | TH16637 |
|  |  | *cmbR*-down | Pr19437/19438 | XhoI |  |  |
| TH17095 | ∆*comX1::*JC1 | *comX1*-up | Pr19417/19419 | XhoI | ST606 | ST606 |
|  |  | *comX1*-down | Pr19420/19421 | XbaI |  |  |

S9 Table: Information for constructions of strains in this study (Continued)

| **Strain ID** | **Genotype** | **PCR amplifications** | | | **Template**  **DNA** | **Parental strains** | |
| --- | --- | --- | --- | --- | --- | --- | --- |
|  |  | **Target sequence** | **Primers** | **Digestion/Fusion** |  |  |  |
| TH17096 | *∆comX1* | *comX1*-up | Pr19422/19424 | XhoI | ST606 | TH17095 | |
|  |  | *comX1*-down | Pr19425/19426 |  |  |  |  |
| TH17098 | *comX2::*JC1 | *comX2*-up | Pr19418/19419 | XhoI | ST606 | ST606 | |
|  |  | *comX2*-down | Pr19420/19421 |  |  |  |  |
| TH17099 | *∆comX2* | *comX2*-up | Pr19423/19424 | XhoI  XbaI | ST606 | TH17098 | |
|  |  | *comX2*-down | Pr19425/19426 |  |  |  |  |
| TH17101 | ∆*comX2-X1::*JC1 | *comX1*-up | Pr19417/19419 | XhoI | ST606 | TH17099 | |
|  |  | *comX1*-down | Pr19420/19421 | XbaI |  |  |  |
| TH17102 | ∆*comX1-X2* | *comX1*-up | Pr19422/19424 | XhoI | ST606 | TH17101 | |
|  |  | *comX1*-down | Pr19425/19426 | XbaI |  |  |  |
| TH17255 | *clpP::*JC1 | *clpP*-up | Pr19648/19649 | XhoI | ST606 | ST606 |  |
|  |  | *clpP*-down | Pr19650/19651 | XbaI |  |  |  |
| TH17256 | ∆*clpP* | *clpP*-up | Pr19648/19649 | XhoI | ST606 | TH17255 |  |
|  |  | *clpP*-down | Pr19650/19651 | XbaI |  |  |  |
| TH17257 | ∆*clpP* *comX2::*JC1 | *comX2::*JC1 | Pr19418/19421 | - | TH17098 | TH17256 |  |
| TH17259 | *rimM::*JC1 | *rimM*-up | Pr19659/19660 | Pr19659/19664 | ST606 | ST606 |  |
|  |  | JC1 | Pr19661/19662 |  | TH8198 |  |  |
|  |  | *rimM*-down | Pr19663/19664 |  | ST606 |  |  |
| TH17258 | *∆clpP comX*^His^ | *comX2*-up | Pr19423/19652 | XbaI | ST606 | TH17257 |  |
|  |  | *comX*^His^ | Pr19653/19654 | XbaI/XhoI | pTH17278 |  |  |
|  |  | *comX2*-down | Pr19425/19426 | XhoI | ST606 |  |  |

S9 Table: Information for constructions of strains in this study (Continued)

| **Strain ID** | **Genotype** | **PCR amplifications** | | | **Template**  **DNA** | **Parental strains** |  |
| --- | --- | --- | --- | --- | --- | --- | --- |
|  |  | **Target sequence** | **Primers** | **Digestion/Fusion** |  |  |  |
| TH17260 | P*_psrA_*-*psrA*^Y247A^ | *bgaA*-up | Pr19705/19711 | Pr19705/19710 | TH15070 | TH8198 |  |
|  |  | *psrA*^Y247A^ | Pr19712/19708 |  | TH6552 |  |  |
|  |  | *bgaA*-down | Pr19709/19710 |  | TH15070 |  |  |
| TH17261 | *∆clpPcomX*^His^*rimM::*JC1 | *rimM::*JC1 | Pr19659/19664 | - | TH17259 | TH17258 |  |
| TH17262 | ∆*thiI::*JC1 | *thiI*-up | Pr19665/19666 | Pr19665/19670 | ST606 | ST606 |  |
|  |  | JC1 | Pr19667/19668 |  | TH8198 |  |  |
|  |  | *thiI*-down | Pr19669/19670 |  | ST606 |  |  |
| TH17263 | ∆*thiI* | *thiI*-up | Pr19665/19671 | Pr19665/19670 | ST606 | TH17262 |  |
|  |  | *thiI*-down | Pr19672/19670 |  |  |  |  |
| TH17264 | ∆*cshA::*JC1 | *cshA*-up | Pr19673/19674 | Pr19673/19678 | ST606 | ST606 |  |
|  |  | JC1 | Pr19675/19676 |  | TH8198 |  |  |
|  |  | *cshA*-down | Pr19677/19678 |  | ST606 |  |  |
| TH17265 | ∆*cshA* | *cshA*-up | Pr19673/19679 | Pr19673/19678 | ST606 | TH17264 |  |
|  |  | *cshA*-down | Pr19680/19678 |  |  |  |  |
| TH17266 | ∆*myy1259::*JC1 | *myy1259*-up | Pr19681/19682 | Pr19681/19686 | ST606 | ST606 | |
|  |  | JC1 | Pr19683/19684 |  | TH8198 |  |  |
|  |  | *myy1259*-down | Pr19685/19686 |  | ST606 |  |  |
| TH17267 | ∆*myy1259* | *myy1259*-up | Pr19681/19687 | Pr19681/19686 | ST606 | TH17266 | |
|  |  | *myy1259*-down | Pr19688/19686 |  |  |  |  |
| TH17269 | ∆*myy450* | *myy450*-up | Pr19689/19695 | Pr19689/19696 | ST606 | TH17268 | |
|  |  | *myy450*-down | Pr19696/19694 |  |  |  |  |

S9 Table: Information for constructions of strains in this study (Continued)

| **Strain ID** | **Genotype** | **PCR amplifications** | | | **Template**  **DNA** | **Parental strains** |
| --- | --- | --- | --- | --- | --- | --- |
|  |  | **Target sequence** | **Primers** | **Digestion/Fusion** |  |  |
| TH17268 | ∆*myy450::*JC1 | *myy450*-up | Pr19689/19690 | Pr19689/19696 | ST606 | ST606 |
|  |  | JC1 | Pr19691/19692 |  | TH8198 |  |
|  |  | *myy450*-down | Pr19693/19694 |  | ST606 |  |
| TH17270 | ∆*myy620::*JC1 | *myy620*-up | Pr19697/19698 | Pr19697/19702 | ST606 | ST606 |
|  |  | JC1 | Pr19699/19700 |  | TH8198 |  |
|  |  | *myy620*-down | Pr19701/19702 |  | ST606 |  |
| TH17271 | ∆*my0620* | *myy620*-up | Pr19697/19703 | Pr19697/19702 | ST606 | TH17270 |
|  |  | *myy620*-down | Pr19704/19702 |  |  |  |
| TH17272 | ∆*bgaA::psrA* | *bgaA*-up | Pr19705/19706 | Pr19705/19710 | TH15070 | TH8198 |
|  |  | *P_psrA_-psrA* | Pr19707/19708 |  | ST606 |  |
|  |  | *bgaA*-down | Pr19709/19710 |  | TH15070 |  |
| TH17273 | P*_hu_*-*psrA* | *bgaA*-up | Pr19705/19711 | Pr19705/19710 | TH15070 | TH8198 |
|  |  | *P_hu_-psrA* | Pr19712/19708 |  | ST606 |  |
|  |  | *bgaA*-down | Pr19709/19710 |  | TH15070 |  |
| TH17274 | AT*::*JC1 | AT-up | Pr19713/19714 | Pr19713/19718 | ST606 | ST606 |
|  |  | JC1 | Pr19715/19716 |  | TH8198 |  |
|  |  | AT-up | Pr19717/19718 |  | ST606 |  |
| TH17275 | 10-AT | 10AT-up | Pr19713/19720 | Pr19713/19718 | ST606 | TH17274 |
|  |  | 10AT-down | Pr19722/19718 |  |  |  |
| TH17277 | 38-AT | 38AT-up | Pr19713/19721 | Pr19713/19718 | ST606 | TH17274 |
|  |  | 18AT-down | Pr19722/19718 |  |  |  |

S9 Table: Information for constructions of strains in this study (Continued)

| **Strain ID** | **Genotype** | **PCR amplifications** | | | **Template**  **DNA** | **Parental strains** |
| --- | --- | --- | --- | --- | --- | --- |
|  |  | **Target sequence** | **Primers** | **Digestion/Fusion** |  |  |
| TH17405 | P*_hu_*-*spxA1* | *bgaA*-up-*P_hu_* | Pr8227/19957 | Pr19705/19710 | TH15070 | TH8198 |
|  |  | *spxA1-bgaA*-down | Pr19958/8230 |  | TH14130 |  |
| TH17279 | pUT18C-TenA | *tenA* | Pr19723/19724 | XbaI/EcoRI | ST606 | DH5α |
| TH17282 | pKT25-RimM | *rimM* | Pr19725/19726 | XbaI/EcoRI | ST606 | DH5α |
| TH17284 | pKT25-ComX | *comX* | Pr19727/19728 | XbaI/EcoRI | ST606 | DH5α |
