# Supplementary material for: The SpxA1-TenA toxin-antitoxin system regulates epigenetic variations of Streptococcus pneumoniae by targeting protein synthesis
Source: PLoS Pathog. 2024 Dec 26;20(12):e1012801. doi: 10.1371/journal.ppat.1012801 (PMC11709252; doi:10.1371/journal.ppat.1012801)
Supplement: Attachment — Submitted filename: Response to Reviewers.docx [file ppat.1012801.s017.docx]

**Responses to Reviewer’s Comments**

**Manuscript No:** PPATHOGENS-D-24-01936

### Title: The SpxA1-TenA Toxin-Antitoxin System Regulates Epigenetic Variations of *Streptococcus pneumoniae* by Targeting Protein Synthesis

**Date:** November 21, 2024

We greatly appreciate the reviewer’s insightful evaluation of our manuscripts, and have revised the entire paper according to the comments by performing additional experiments and modifying the text/figures. With these valuable suggestions, the revised manuscript has been significantly improved. The revised manuscript has been submitted as requested, and the changes are highlighted in the revised manuscript. The followings are my responses to the review comments.

**Editor:**

**In Fig. S4A and B. the ∆spxA2 exhibits increased spxA1 transcription and also a modest but significant decrease in hsdSA1mRNA. In my reading, these findings are not reflected in the text, lines 236-239. In addition, I believe that there should be “∆’’ placed before "spxA1rev" and spxA2rev" in Fig. S4A. (****Note also that I believe that Fig. S4 indicates that you already constructed a spxA1-complemented strain that Reviewer 3 suggested to include in order to reinforce the conclusions of Fig. 4B.)**

We apologize for the inadequate description of these results. We have revised the text according to the suggestions:

1. The increased transcription of *spxA1* in ∆*spxA2* is elaborated in the revised Results section (lines 271-276, page 10).

2. We have added the “∆” symbol before “*spxA1*^rev^” and “*spxA2*^rev^” in the revised **Fig. S4A**.

3. We have performed new experiments to detect *tenA* transcription in the *spxA1*-complemented strain. The result showed that the reduced transcription in the *spxA1* deletion mutant was restored to the parental level in its revertant strain (revised **Fig 4B**) (lines 256-260, page 9). These data further support our conclusions and have been added to the revised manuscript.

**Major Issues**

**Reviewer #1:**

None

**Reviewer #2:**

None

**Reviewer #3:**

**1.** **In lines 256-258 the authors state their findings confirm that TenA is transcriptionally repressed by SpxA1.** **How do the authors think this is happening? Is this just because the proteins are on the same transcript? To explore this finding, it would be useful for the authors to show a knock in of spxAI in a neutral locus represses tenA transcription levels (see Fig4B). Regardless of this experiment, Fig4B needs a spXAI** **complemented control.**

We thank the reviewer for pointing out this important issue. We have performed new experiments and revised the text accordingly to strength our conclusion that SpxA1 represses the transcription of the *tenA-spxA1* operon*.*

1. We performed a new experiment to assess the transcriptional impact of SpxA1 on the *tenA-spxA1* operon by overexpressing *spxA1* under the control of the *hu* promoter (one of the strongest promoters in *Streptococcus pneumoniae*) in a neutral *bgaA* locus. This locus has been widely used as neutral locus for *in trans* gene expression in this bacterium. Our RT-qPCR result demonstrated that the *tenA* transcription was significantly repressed in the *spxA1* overexpression strain as compared with that of the JC1 control. This result is shown in the new figure **Fig 4C** (lines 260-266, page 9).

2. We conducted a genetic complementation experiment to confirm the causal relationship between SpxA1 and *tenA* transcription by knock in *spxA1* in the *spxA1* locus of the ∆*spxA1* strain, which is referred to as ∆*spxA1^rev^*. The *in situ* restoration of *spxA1* reverted the level of the *tenA* mRNA to that of the wild type strain. This result is shown in the new figure **Fig 4B** (the last bar) (lines 256-260, page 9).

**Minor Issues**

**Reviewer #1:**

None

**Reviewer #2:**

**1. Line 469: The journal may not allow “****Data not shown”. If you have the data, it should go in supplement. Alternatively, delete this sentence.**

We have deleted this sentence (line 505, page 17).

**2.** **Line 490: With respect to the sentence “In particular, the vast majority of the protein hits in the TenA affinity pull-down were associated with protein synthesis.”,** **is it possible that TenA actually only binds RimM, but RimM is bound to these other ribosomal proteins?**

We believe that RimM binds to other ribosomal proteins based on the abundance of many ribosomal proteins in the pulldown experiment data. Of course, it is also likely that TenA only binds RimM. Since the main feature of the pull-down assay is extensive screening of all the directly and indirectly interacting proteins, our results cannot distinguish the following two possibilities of TenA’s working module on protein synthesis: TenA interacts with RimM as well as other ribosomal proteins or TenA only binds RimM to further impact the function of the holoenzyme. We have added this point in the revised Discussion section (lines 528-530, page 17).

**3. Table 1. Is a p value of zero a legitimate value to report? I believe it is better to report p < [smallest representable value].**

Revised.

**4. Delete “****interestingly” here and elsewhere (line 295). That is opinion and is not very scientific.**

Revised (line 35, page 2; line 332, page 11).

**5. Line 121: Change “****insertion sequence” to “inserted sequence”. An insertion sequence is technically a transposable element in bacteria.**

Revised (lines 148 and 154, page 6).

**6. Line 148: Delete “finally”.**

Revised (line 175, page 7).

**7. Lines 158, 162, 341, 452:** **Why is “****via” italicized? It shouldn’t be.**

Revised (lines 185 and 189, page 7; line 376, page 13; line 487, page 16).

**8. Line 239: I believe you want to delete “****as SpxA1” at the end of the sentence. Otherwise, rewrite the sentence to be more precise.**

Revised (line 275, page 10).

**9. Line 256: “have confirmed” is a little too strong. I suggest changing to “****suggest” or “indicate”.**

Revised (lines 293, page 10).

**10. Line 264: Change “trial” to “experiment”.**

Revised (line 301, page 11).

**11. Line 304: Start sentence with “This”.**

Revised (line 341, page 12).

**12. Line 319: Change “mostly” to “****most”.**

Revised (line 355, page 12).

**13. Line 346: Change “persisted robustly” to “abundant”, or something more clear.**

Revised (line 383, page 13).

**14. Line 360: Change “TIRGR4” to “TIGR4”.**

Revised (line 395, page 13).

**15. Line 378: Strain not “stain”.**

Revised (line 414, page 14).

**16****. In the RNA isolation and sequencing section in Materials and Methods, we rRNA removed? This wasn’t mentioned. If not, then fine.**

This issue is elaborated in the revised Materials and Methods section (lines 630-635, page 21).

**17. Line 657: Delete “and vitality”.**

Revised (line 699, page 23).

**Reviewer #3:**

**a. Fig1A: Is the arrow in the bottom right panel supposed to be blue? The colony looks opaque.**

Revised in **Fig1A**.

**b. What does the red arrow mean in Fig2B.**

Revised in the figure legend.

**c. Fig5E lacks a Y axis label**

The Y axis labels were added in **Fig 3B** and **Fig 5E**.
